# Convergence between light quality and methyl jasmonate signaling in the regulation of betalain biosynthesis

**DOI:** 10.3389/fpls.2026.1865544

**Published:** 2026-06-26

**Authors:** Hui Wang, Chao Yang, Yanling Yu, Dayou Cheng, Cuihong Dai, Chengfei Luo

**Affiliations:** 1 School of Chemistry and Chemical Engineering, Harbin Institute of Technology, Harbin, China; 2 School of Astronautics, Harbin Institute of Technology, Harbin, China; 3 School of Medicine and Health, Harbin Institute of Technology, Harbin, China

**Keywords:** betalain, HY5, light quality, MeJA, MYB, MYC2

## Abstract

Betalains are nitrogen-containing pigments that produce red-violet and yellow-orange coloration in plants of the order Caryophyllales. Owing to their antioxidant, anti-inflammatory and anticancer activities, they have attracted considerable interest as natural food colourants and functional ingredients. Betalain biosynthesis is governed by both developmental programmes and environmental inputs, among which light quality and jasmonate signalling are key regulators. This review systematically examines the independent and synergistic roles of these two signals in controlling betalain production. Exogenous methyl jasmonate (MeJA), a widely used elicitor, is converted intracellularly to the bioactive ligand JA-Ile, which triggers SCF(COI1)-dependent degradation of JAZ repressors, thereby releasing MYC2 and MYC3 to activate downstream transcription. Light quality, operating through specific photoreceptors and the COP1-HY5 module, modulates betalain accumulation in a wavelength- and species-specific manner. Accumulating evidence suggests that both pathways converge on R2R3-MYB transcription factors, which bind MBS motifs in the promoters of the key structural genes CYP76AD1 and DODA. The betalain biosynthetic pathway proceeds through four principal enzymatic steps to yield betacyanins and betaxanthins, catalysed sequentially by ADH, CYP76AD1, DODA and cDOPA5GT. We propose a MYC2-HY5-MYB regulatory axis as a working model for the coordinate regulation of betalain biosynthesis, and identify key questions that remain to be resolved through functional studies in betalain-producing species.

## 1 Introduction

Betalains are water-soluble nitrogen-containing pigments restricted to the order Caryophyllales. They comprise two structural classes: red-violet betacyanins and yellow-orange betaxanthins, both derived from the common chromophore betalamic acid (Gandía-Herrero and García-Carmona, 2013). As natural colourants (E162), betalains are employed in dairy products, confectionery and processed foods as alternatives to synthetic red pigments (Ans E.P.O.F, 2015; Sigwela et al., 2022). Beyond their colouring function, they exhibit antioxidant, anti-inflammatory and anticancer activities (Khan, 2016; Milech et al., 2023; Wadi and Hakeem, 2025), and recent advances in extraction and stabilization technologies have broadened their potential for industrial application (Azwanida Zakaria et al., 2025). Global sugar beet production exceeds 310 million tonnes per year, with approximately 30% of processing by-products amenable to pigment extraction through green technologies, underscoring the economic incentive to optimize betalain production (Soare et al., 2021).

The betalain biosynthetic pathway originates from tyrosine. Tyrosine is hydroxylated to L-DOPA by CYP76AD family cytochrome P450 enzymes, and DOPA-4,5-dioxygenase (DODA) cleaves L-DOPA to yield betalamic acid. This central intermediate diverges into two branches: condensation with cyclo-DOPA produces betacyanins, while spontaneous condensation with amino acids or amines yields betaxanthins (Tossi et al., 2021). The pathway is subject to multi-level regulation by both internal developmental programmes and external environmental signals (Sadowska-Bartosz and Bartosz, 2021).

Jasmonate signalling is a central regulator of plant secondary metabolism. Experimentally, methyl jasmonate (MeJA), a volatile methyl ester of jasmonic acid, is the most commonly used exogenous elicitor owing to its membrane permeability and stability. Upon cellular uptake, MeJA is demethylated to jasmonic acid and subsequently conjugated to isoleucine by JASMONATE RESISTANT 1 (JAR1), yielding the canonical bioactive jasmonate, JA-Ile (Ghorbel et al., 2021). JA-Ile binds the SCF(COI1) E3 ubiquitin ligase complex, triggering ubiquitin-dependent proteasomal degradation of JAZ transcriptional repressors, which releases MYC2 and MYC3 from inhibition (Chini et al., 2007). In parallel, light quality, perceived by the photoreceptors phytochrome B, cryptochromes and UVR8, is integrated through the COP1-HY5 module. Light disrupts COP1/SPA activity, stabilizing HY5 and enabling it to regulate photomorphogenesis and secondary metabolism (Ouzounis et al., 2015).

These two signalling pathways are not independent. ChIP-seq and EMSA analyses in Arabidopsis have shown that MYC2 and MYC3 directly bind PBE elements in the HY5 promoter to activate its transcription (Ortigosa et al., 2020; Song et al., 2022), establishing a molecular connection between jasmonate and light signalling at the level of transcriptional regulation. Both MYC2/MYC3 and HY5 are proposed to converge on R2R3-MYB transcription factors, which serve as the central regulators of betalain biosynthesis in Caryophyllales. These MYB factors bind MBS (MYB-binding site) elements in the promoters of CYP76AD1 and DODA (Hatlestad et al., 2015; Xue et al., 2025). However, direct experimental evidence for this convergence is currently confined to Arabidopsis, and whether the MYC2-HY5-MYB regulatory axis operates in betalain-producing species remains to be determined.

**Figure 1:**
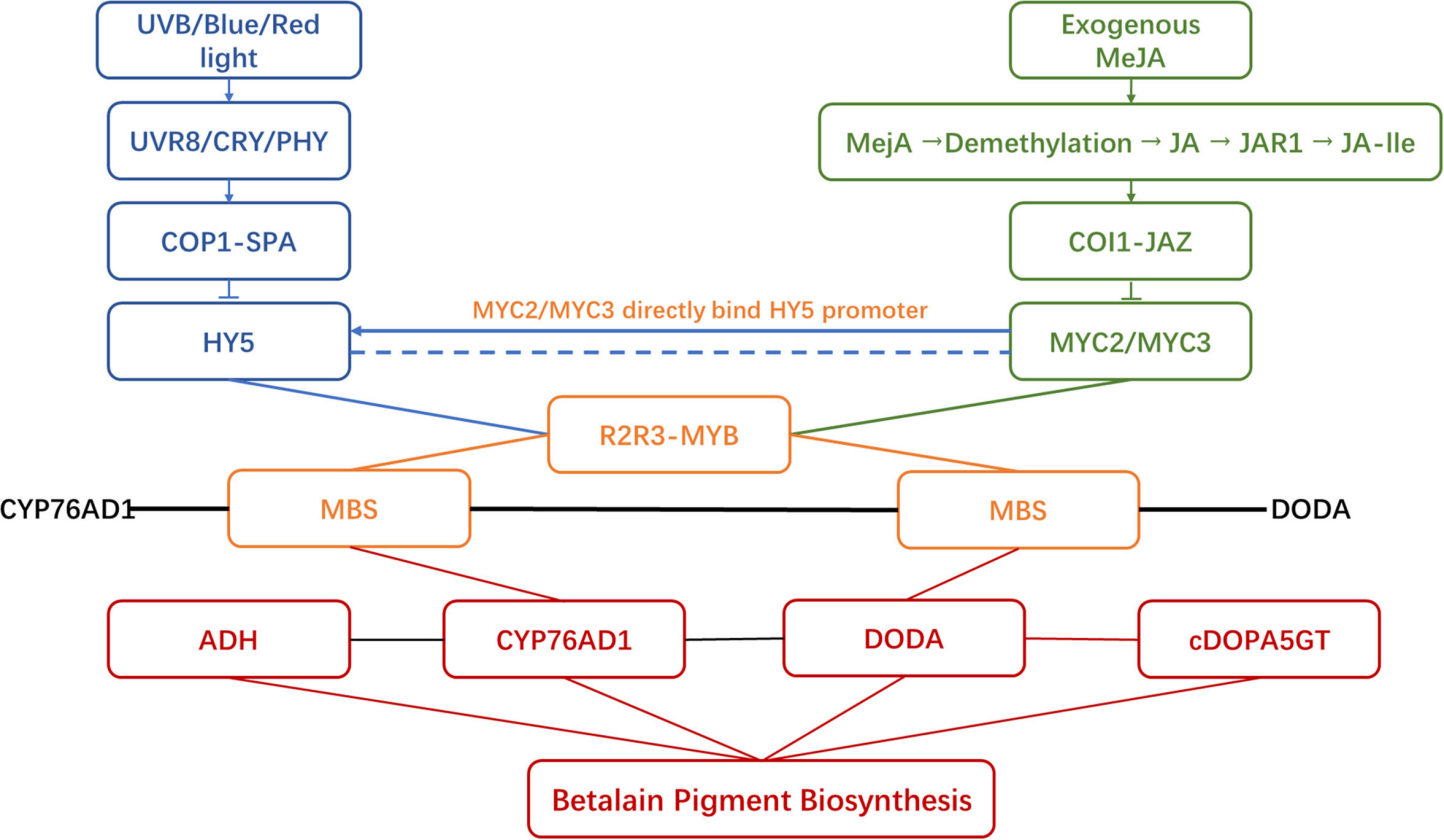
Integrated model of MeJA and light quality signal crosstalk regulating betalain biosynthesis. Light quality signals (UV-B, blue, or red light) are perceived by specific photoreceptors (UVR8, CRY1/CRY2, and PHYB, respectively) and converge on the COP1-SPA complex. Light disrupts COP1-SPA activity, relieving COP1-mediated degradation of HY5 and allowing its nuclear accumulation. On the jasmonate side, exogenous MeJA is demethylated to JA and conjugated to isoleucine by JAR1 to produce the bioactive ligand JA-Ile, which promotes SCF(COI1)-mediated ubiquitination and proteasomal degradation of JAZ repressors, thereby releasing MYC2 and MYC3 (Chini et al., 2007). MYC2 and MYC3 directly bind the HY5 promoter at PBE elements (Ortigosa et al., 2020), establishing a primary transcriptional link between the two pathways. A potential feedback loop from HY5 to MYC2/MYC3 is hypothesized but awaits experimental validation (Song et al., 2022). Both signals converge on R2R3-MYB transcription factors, which directly bind MBS elements in the promoters of CYP76AD1 and DODA (Hatlestad et al., 2015; Xue et al., 2025). The betalain biosynthetic pathway proceeds through four key enzymes (ADH, CYP76AD1, DODA, cDOPA5GT) to produce betalain pigments. Solid arrows indicate experimentally supported regulatory relationships; dashed arrows indicate hypothesized interactions; T-bars indicate inhibition or degradation. COP1, CONSTITUTIVE PHOTOMORPHOGENIC 1; SPA, Suppressor of PhyA-105; HY5, ELONGATED HYPOCOTYL 5; CRY, cryptochrome; PHYB, phytochrome B; UVR8, UV RESISTANCE LOCUS 8; MeJA, methyl jasmonate; JA, jasmonic acid; JA-Ile, jasmonoyl-isoleucine; JAR1, JASMONATE RESISTANT 1; SCF, Skp1-Cullin-F-box; COI1, CORONATINE INSENSITIVE 1; JAZ, JASMONATE ZIM-DOMAIN; MYC2, bHLH transcription factor; PBE, MYC2-binding element; MBS, MYB-binding site; ADH, arogenate dehydrogenase; CYP76AD1, cytochrome P450 76AD1; DODA, DOPA-4,5-dioxygenase; cDOPA5GT, cyclo-DOPA 5-O-glucosyltransferase.

Previous reviews have addressed the betalain biosynthetic pathway (Sadowska-Bartosz and Bartosz, 2021; Tossi et al., 2021) or the roles of jasmonates in plant defence (Ghorbel et al., 2021) as separate topics. No review has systematically examined the crosstalk between light quality and jasmonate signalling in the specific context of betalain regulation. Here we integrate evidence from JA signalling, light signalling and betalain-specific transcriptional regulation, and propose a MYC2-HY5-MYB signal integration model (**Figure 1**) as a framework to guide future experimental investigation in Caryophyllales.

## 2 Environmental and hormonal signals in plant pigment synthesis

### 2.1 Chemical properties and economic value of betalains

**Figure 2:**
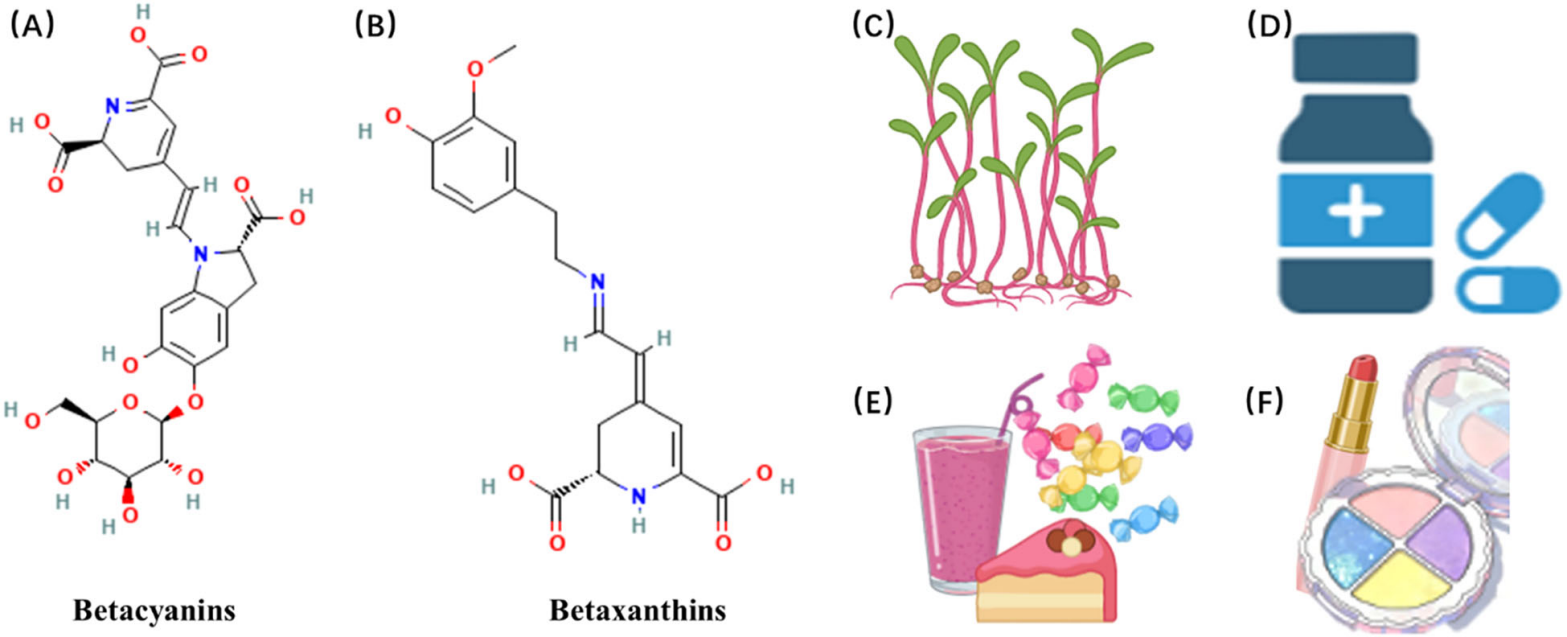
Chemical structures and application domains of betalains. **(A)** Representative betacyanin. **(B)** Representative betaxanthin. **(C)** Beet seedlings. **(D–F)** Applications in food, pharmaceuticals, and cosmetics.

As introduced above, betalains are water-soluble nitrogen-containing pigments with well-documented antioxidant, anti-inflammatory, and anticancer activities. Beyond their role as natural food colorants (E162), their functional properties have driven expanded applications in functional foods and sports nutrition, including high-protein biscuits, probiotic beverages, and sports supplements. Global sugar beet production exceeds 310 million tonnes annually, with approximately 30% of processing by-products amenable to valorization through green extraction technologies, reducing resource waste (Soare et al., 2021). **Figure 2** provides an overview of betalain chemical structures and their industrial and nutraceutical applications.

### 2.2 Betalain biosynthetic pathway

Betalain biosynthesis begins with tyrosine produced via the shikimate pathway. The amino group of this precursor is provided by glutamine transamination, with phosphoenolpyruvate (PEP) and erythrose-4-phosphate (E4P) condensing to form chorismate, ultimately catalyzed by prephenate dehydrogenase (PDH). Tyrosine is first hydroxylated to L-DOPA in the cytoplasm and endoplasmic reticulum by tyrosine hydroxylase (TYRH; recent studies confirm that CYP76AD family cytochrome P450 enzymes possess this activity) (Sunnadeniya et al., 2016). Subsequently, 4,5-DOPA dioxygenase (DODA) catalyzes aromatic ring cleavage to generate 4,5-seco-DOPA, which spontaneously cyclizes to form betalamic acid. This key intermediate enters two branching pathways to form different pigments: condensation with cyclo-DOPA (generated from L-DOPA oxidation catalyzed by CYP76AD1) produces red betacyanins, while spontaneous condensation with amino acids/amines yields yellow betaxanthins (Tossi et al., 2021).

**Figure 3:**
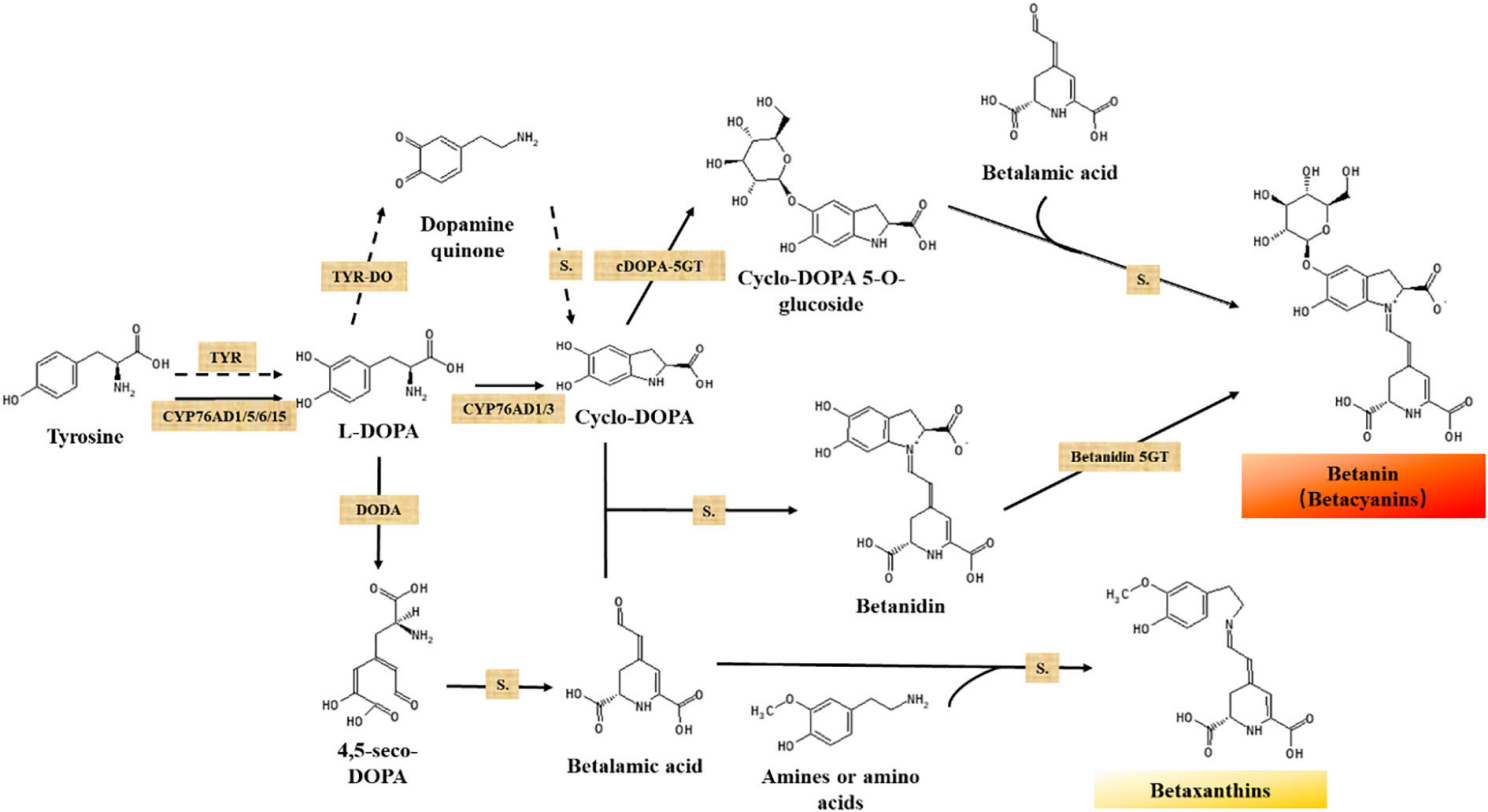
Betalain biosynthetic pathway. This figure was drawn by the authors based on published betalain biosynthetic pathway information (Sunnadeniya et al., 2016; Tossi et al., 2021; Xie et al., 2021).

This biosynthetic system is subject to multi-level regulation: in light signaling, red light regulates DHS (3-deoxy-D-arabino-heptulosonate-7-phosphate synthase) gene expression or affects metabolic substrate supply through the PhyB-PIF4 module, indirectly modulating carbon flux from the shikimate pathway toward aromatic compound synthesis (Xu and Zhu, 2021; Yokoyama et al., 2022). Blue light acts synergistically through the CRY1-HY5 pathway, while UV-B promotes nitrogen assimilation by activating HY5 to enhance nitrate transporter NRT2.1 expression. The rate-limiting enzyme CYP76AD1 and glycosyltransferases are directly regulated by transcription factors such as MYB, while integrating hormone signals including cytokinins and ethylene, as well as environmental temperature changes, forming a finely regulated synthetic system with coordinated nitrogen metabolism and light-hormone networks. Following synthesis, pigments undergo glycosylation catalyzed by cyclo-DOPA-5-O-glucosyltransferase (cDOPA5GT) and betanidin-5-O-glucosyltransferase (Betanidin 5GT), and are ultimately stored in vacuoles (Sadowska-Bartosz and Bartosz, 2021). **Figure 3** summarizes the betalain biosynthetic pathway with emphasis on regulatory steps relevant to this review.

## 3 Methyl jasmonate (MeJA) signal transduction and function

Jasmonates comprise a family of related compounds, including jasmonic acid (JA), its methyl ester (MeJA), and the amino acid conjugate JA-isoleucine (JA-Ile), among others. Among these, MeJA is the most widely used exogenous elicitor in studies of plant secondary metabolism due to its membrane permeability and volatility, which facilitate efficient cellular uptake. Once inside the cell, MeJA is demethylated to JA and subsequently conjugated to isoleucine by JAR1 to form the bioactive signal JA-Ile (Ghorbel et al., 2021). This section reviews the MeJA signaling pathway and its physiological functions.

### 3.1 MeJA signaling pathway and physiological functions

Under these stress conditions, MeJA and related compounds are rapidly activated and accumulated, subsequently initiating a series of defence measures (Ghorbel et al., 2021; Ma et al., 2025). The physiological functions of MeJA are primarily achieved through its core signal transduction module, COI1-JAZ-MYC. When plants are challenged by pests, pathogens, or stress conditions, biologically active jasmonoyl-isoleucine (JA-Ile) is first synthesized. Subsequently, JA-Ile is recognized by the SCFCOI1 E3 ubiquitin ligase complex and binds to JAZ proteins, forming a hormone-receptor complex that triggers ubiquitination of JAZ repressor proteins and their degradation via the proteasome pathway. Degradation of JAZ proteins relieves the inhibition of core transcription factors (such as MYC2); these released MYC transcription factors then activate the transcription and expression of numerous defense-related genes (Milech et al., 2022; Wang et al., 2023).

**Figure 4:**
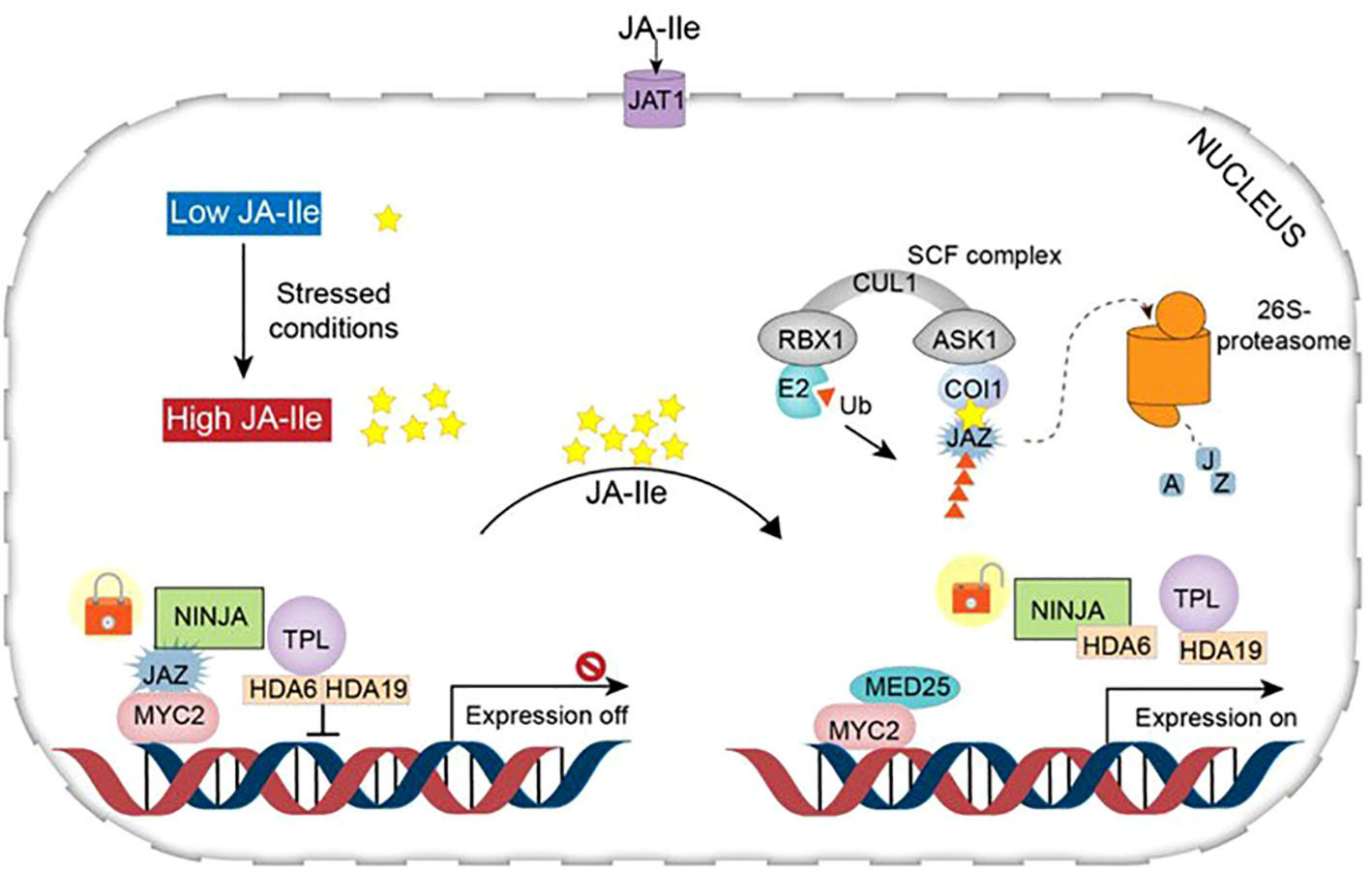
Illustrates the working model of the COI1-JAZ-MYC jasmonate signal transduction module (Ali and Baek, 2020; Wang et al., 2021).

**Figure 5:**
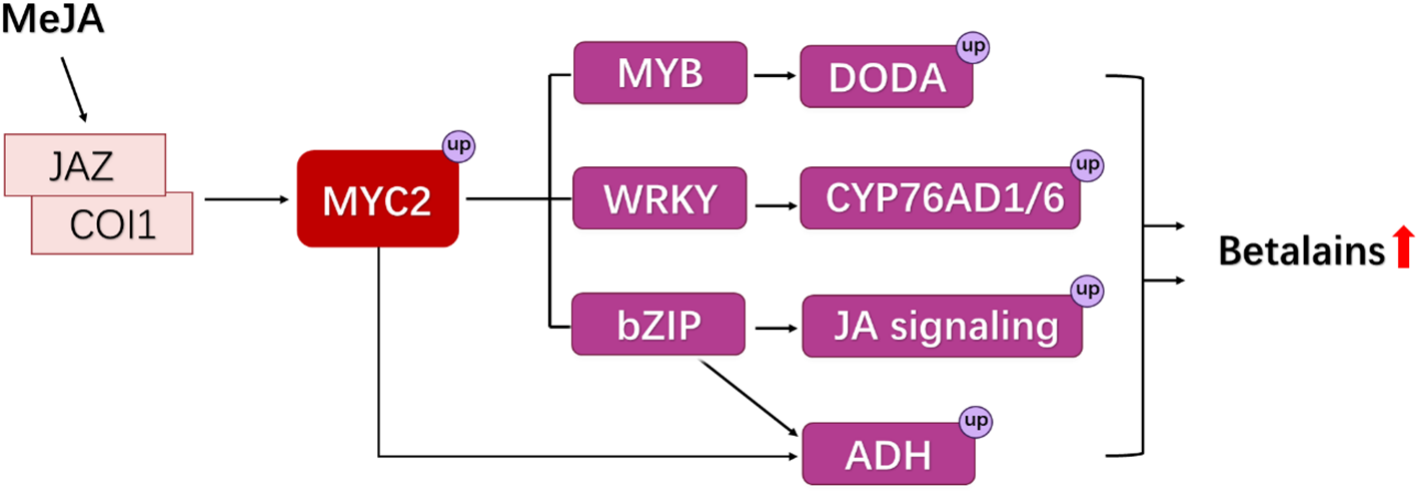
Mechanistic model of MeJA signaling pathway regulation of betalain synthesis genes. This figure was drawn by the authors based on published betalain biosynthetic pathway information (Milech et al., 2022, Milech et al., 2023; Hamzah et al., 2024).

Jasmonates thus serve dual roles: activating defence against herbivores and pathogens, and modulating growth and development. The key mediators of this signaling pathway are MYC transcription factors, which are inhibited by JAZ proteins under resting conditions. In the presence of active jasmonates, JAZ proteins instead bind to COI1, serving as co-receptors in forming the hormone perception complex. This process ultimately leads to degradation of JAZ repressors and restoration of MYC protein transcriptional activity, thereby initiating downstream responses, **Figure 4** illustrates the canonical JA signaling module as mechanistic background for the betalain-specific model presented in **Figure 5**. Through this core module, MeJA broadly regulates plant physiological functions: activating defense responses by directly inducing synthesis of pathogenesis-related proteins and protease inhibitors while driving biosynthesis of defensive secondary metabolites, thereby enhancing resistance to pathogens and insects (Ji et al., 2021a); alleviating oxidative damage by increasing antioxidant enzyme activities to effectively scavenge excess reactive oxygen species and protect cell membrane structure and function (Wang et al., 2024); and promoting secondary metabolism, particularly in betalain synthesis, where jasmonate signalling upregulates the expression of key enzyme genes, including tyrosine hydroxylase and DOPA dioxygenase, through transcription factor activation, while promoting precursor tyrosine supply, thereby significantly enhancing betacyanin and betaxanthin accumulation (Xie et al., 2021; Milech et al., 2022).

While MeJA signaling also intersects with other phytohormone pathways (Ghorbel et al., 2021; Zhao et al., 2024), the interaction between MeJA and light quality signals is of particular relevance to betalain biosynthesis, as both signals converge on shared transcription factors that directly regulate betalain structural genes. This crosstalk will be examined in Section 6.

### 3.2 Phenotypic effects of MeJA-driven pigment accumulation

MeJA, as a key exogenous signal molecule in plants, can systematically induce betalain synthesis and accumulation in various plant species. Based on comprehensive research (summarized in **Table 1**), its inductive effects exhibit significant time and dose dependence, influenced by both species and treatment methods. Across different physiological systems, exogenous MeJA demonstrates efficient inducing capacity. In hydroponic and cell suspension cultures of herbaceous plants, moderate to low concentrations (10-100 µM) significantly increase pigment content within days to weeks. For example, in hydroponic systems of *Alternanthera philoxeroides* and *A. sessilis*, 100 µM MeJA treatment increased total betalain content by up to 70.05% and 76.30%, respectively (Milech et al., 2023). Foliar application (1000 ppm) during late fruit development in dragon fruit increased pulp betacyanin content by approximately 29.9% (Hamzah et al., 2024). Notably, in cultured *Celosia argentea* cells, approximately 50 µM MeJA addition rapidly (4–6 days) increased betacyanin production by 33.3% to 3.93-fold (Mueangnak et al., 2025; Sang A Roon et al., 2025), highlighting its application potential in large-scale biosynthesis.

**Table 1:** External MeJA treatment on acumulation of beetroot pigments in different plants.

Species	Concentration	Treatment method	Treatment time	Effect (betalain increase)	Reference
*Alternanthera philoxeroides*	100 µM	Added to hydroponic medium	2, 4, 7, 15 days	Total: 46.1%, 48.1%, 70.1%, 56.1%	(Milech et al., 2023)
*Alternanthera sessilis*	100 µM	Added to hydroponic medium	2, 4, 7, 15 days	Total: 30.1%, 54.1%, 60.1%, 76.3%	(Milech et al., 2023)
*Hylocereus polyrhizus*	1000 ppm	Foliar spray (15 and 22 DAF)	Harvested 35 days after spraying	Pulp betacyanin: +29.9%	(Hamzah et al., 2024)
*Amaranthus mangostanus*	10 µM	Seed germination in MeJA buffer	4 days	Betacyanin: 12.2× (dark), 4.3× (light)	(Cao et al., 2012)
*Celosia argentea*	49.97 µM	Added to cell suspension culture (day 9)	6 days after addition	Total betacyanin: 139.99 mg/L (3.93×)	(Sang A Roon et al., 2025)
*Celosia argentea*	50 µM	Added to cell suspension culture (day 8)	4 days after addition	Betacyanin: 3.32 mg/g DW (+33.3%)	(Mueangnak et al., 2025)
*Amaranthus tricolor*	100 µM	Spray on *AtrDODA1* promoter-transfected tobacco leaves	3, 6, 12, 24 h	Promoter activity: strongest at 12–24 h	(Xie et al., 2025b)
*Alternanthera sessilis*	100 µM	Added to hydroponic nutrient solution	48 h (transcriptome); 96 h (pigment)	Betacyanin: +20%; upregulation of secondary metabolic pathways	(Milech et al., 2022)

From a molecular perspective, MeJA primarily functions through transcriptional regulation, although its regulatory network may exhibit species specificity. Studies in *Amaranthus tricolor* demonstrated that MeJA directly positively regulates the key synthesis gene *AtrDODA1–1* by activating JA-responsive elements in its promoter (Xie et al., 2025a). However, comparative studies of two closely related Alternanthera species revealed a more complex picture: in *A. philoxeroides*, MeJA treatment upregulated expression of key genes including *ADH* and *DODA*; whereas in *A. sessilis*, despite significant pigment accumulation, expression of related synthesis genes was generally suppressed (Milech et al., 2023). This difference suggests that beyond typical transcriptional activation pathways, MeJA may influence betalain accumulation in certain species through unique post-transcriptional regulation or negative feedback mechanisms.

Collectively, these studies establish exogenous MeJA as a potent elicitor of betalain synthesis, although its mode of action varies across species and tissues, and the upstream and downstream regulatory pathways require further investigation.

### 3.3 Molecular mechanisms of MeJA regulation

Transcriptome analysis of MeJA-elicited *Alternanthera sessilis* identified the upregulation of transcription factors from the MYB, WRKY, and bZIP families, suggesting their involvement in MeJA-mediated betalain biosynthesis (Milech et al., 2022). In *A. philoxeroides*, MeJA treatment upregulated the expression of ADH, CYP76AD1, and DODA, with total betalain content increasing by 30-76% depending on treatment duration (Milech et al., 2022, Milech et al., 2023). Based on these transcriptomic and phenotypic observations, combined with the demonstrated role of MYB factors as direct activators of CYP76AD1 and DODA promoters (Hatlestad et al., 2015), a working model of MeJA-elicited betalain regulation is presented in **Figure 5**.

In red-fleshed dragon fruit, preharvest MeJA application significantly increased pulp betacyanin content, total phenolics, flavonoids, and antioxidant activity (Hamzah et al., 2024), indicating that MeJA can enhance betalain accumulation and associated nutritional quality across diverse betalain-producing species (Xie et al., 2025a).

## 4 Regulatory roles and mechanisms of light quality

### 4.1 Photoreceptors and downstream signaling networks

Plant photoreceptors play central roles in regulating secondary metabolism, coordinating bidirectional communication between the nucleus and chloroplasts. Through forward signalling, photoreceptors control chloroplast development and photosynthesis-related gene expression. Through retrograde signalling, they receive and integrate signals from chloroplast metabolic status, including tetrapyrroles and the isoprenoid intermediate MEcPP, forming a dynamic feedback loop that balances growth and defence (Griffin and Toledo-Ortiz, 2022). Within this framework, different light qualities initiate fine regulation of secondary metabolism through specific receptors: UVR8 specifically perceives UV-B radiation, significantly promoting phenolic, flavonoid, and anthocyanin synthesis by activating the HY5 transcription factor and downstream phenylpropanoid pathway key enzymes, including phenylalanine ammonia-lyase (PAL) and chalcone synthase (CHS) (Wu et al., 2025). Blue light activates the core transcription factor HY5 through cryptochromes (CRY), forming a regulatory hub. This CRY-HY5 module, by stabilizing HY5 protein and regulating its activity, initiates key processes including stress responses (e.g., high light adaptation and cold acclimation), pigment synthesis, and cell wall development under different physiological scenarios, thereby integrating light signals to coordinate multidimensional environmental adaptation and growth and development in plants (Li et al., 2021a; Ashikhmin et al., 2024; Hwang et al., 2024).

**Figure 6:**
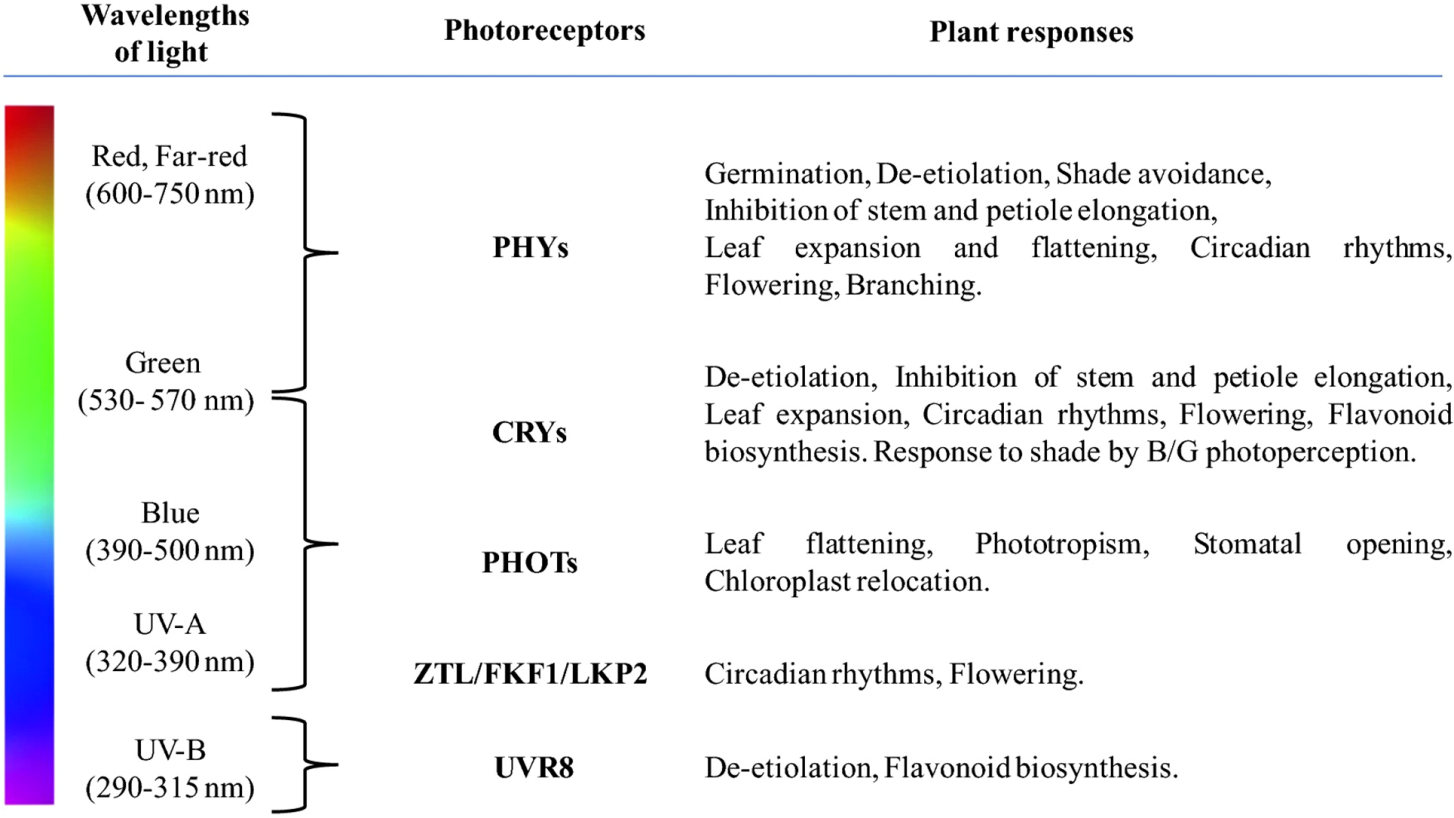
Spectral wavelength specificity of the main plant photoreceptors and related plant photomorphogenesis responses. Phytochromes (PHYs), cryptochromes (CRYs), phototropins (PHOTs), Zeitlupe family proteins (ZTL/FKF1/LKP2), and UV resistance Locus 8 (UVR8) (Paradiso and Proietti, 2022).

Phytochromes (PHYs), as red/far-red receptors, regulate the COP1/HY5 core module. Under red light, activated PHYB interacts with SPA, inhibiting COP1/SPA activity, stabilizing HY5, and promoting photomorphogenesis; under far-red light, SPA nuclear entry enhances COP1-mediated HY5 degradation, inhibiting photomorphogenesis. Phytochrome signaling also cross-talks with JA and SA pathways, regulating development, stress responses, and adaptation (Bhatnagar et al., 2020). Green light, although less absorbed by chloroplasts, downregulates blue light receptors CRY2 and PHOT2, antagonizing blue light signaling and promoting photooxidation-resistant metabolites (e.g., proanthocyanidins B2/B3); it also activates JA/SA defense-related genes, enhancing secondary metabolic defense capacity (Zheng et al., 2019). Notably, photoreceptor signals converge on HY5, which serves as a key integration node for chloroplast retrograde signals (e.g., GUN1, MEcPP pathways). Thus, with HY5 as a hub, a multi-layered signaling network forms, responding to external light while receiving internal chloroplast feedback, precisely balancing resource allocation between photosynthetic primary metabolism and defensive secondary metabolism (**Figure 6**). **Figure 6** provides an overview of photoreceptor spectral specificity and downstream photomorphogenesis responses (Paradiso and Proietti, 2022).

### 4.2 Specific regulation of betalain synthesis by different light qualities

Light quality is a key environmental factor regulating betalain synthesis, but its effects exhibit pronounced species, tissue, and even cultivar specificity (Reis et al., 2015; Xie et al., 2025b). To systematically present the specific effects of different light quality treatments on betalain synthesis, major research findings are summarized in **Table 2** below.

**Table 2:** Summary of different light quality treatment effects on betalain biosynthesis.

Light quality	Light intensity (μmol·m^-^²·s^-^¹)	Photoperiod (L/D, h)	Plant material	Key findings (betalain accumulation)	Reference
B, R, RB, BFR, RFR, W	50	16/8	*Beta vulgaris* L. hairy roots	BFR: betacyanin 4.2× R; highest betacyanin/betaxanthin ratio	(Boo et al., 2002)
W, B, R	W:25, B:12, R:22	16/8	*Alternanthera brasiliana* calli	B: highest betaxanthin (12×) and glycosylated betacyanin (4.5×); R: minimal induction	(Reis et al., 2018)
W, B, R	W:25, B:12, R:22	16/8	Four *Alternanthera* spp. whole plants	W/B: effective for *A. brasiliana*; R: effective for *A. philoxeroides*; B: strongest for flavonoids	(Reis et al., 2015)
Composite LED (R/B/W)	150.2 ± 5.4	14/10	*Beta vulgaris* L.	High R ratio: most significant promotion of betalains	(Oh et al., 2022)
W, R, B	W:80, R:40, B:2	16/8	*Suaeda salsa* L. *calli*	W > R > B for betalain promotion; higher intensity increases content	(Zhao et al., 2010)
B, R	300	16/8	*Amaranthus cruentus*	Significant betalain accumulation; enhanced insect resistance	(Portillo-Nava et al., 2021)
UV-A, UV-B	500 μE	18/6	*Mesembryanthemum crystallinum*	Significant increase in betalain accumulation	(Vogt et al., 1999)
UVB	6.4 W·m^-^²	–	*Opuntia ficus-indica*	Significant increase in betalain accumulation	(Ortega-Hernández et al., 2020)

B, blue light (470 nm); R, red light (660 nm); RB, red+blue light; BFR, blue+far-red light; RFR, red+far-red light; W, white light (full spectrum); LED, light-emitting diode; UV-A, ultraviolet A light (315–400 nm); UV-B, ultraviolet B light (280–315 nm); μE, microeinstein; –, not specified. Light intensity units are μmol·m^-^²·s^-^¹ unless otherwise specified.

Overall, blue, white, and UV light are effective inducers in most cases, while red light effects range from strong inhibition to significant promotion, reflecting regulatory complexity. For example, in *Alternanthera brasiliana*, blue light induced betalain synthesis far exceeding white light, whereas red light severely inhibited pigment accumulation (Reis et al., 2018). In red beet hairy roots, blue+far-red (BFR) light most effectively promoted betacyanin accumulation (4.2-fold higher than red light alone), while monochromatic red light inhibited synthesis (Boo et al., 2002). UV-B, as an environmental stress signal, induces betalain accumulation in various species (e.g., Amaranthus) by activating UVR8 signaling, often associated with reactive oxygen species accumulation (Polturak and Aharoni, 2018; Ortega-Hernández et al., 2020). However, in red beet calli (El-Ashry et al., 2020), *Alternanthera philoxeroides* (Reis et al., 2015), and vertical farm-cultivated table beets, red light or high red-light ratios most effectively promoted betalain accumulation (Oh et al., 2022). Specific light combinations often surpass monochromatic light effects, e.g., BFR promoting betacyanin in *A. brasiliana* (Boo et al., 2002) and blue/red light inducing pigment accumulation with enhanced insect resistance in grain amaranth (Portillo-Nava et al., 2021). This suggests that precise light quality configuration in artificial environments can directionally optimize betalain yield and composition in target plants.

In summary, light quality regulation of betalain synthesis presents a diversified picture, strongly suggesting underlying complex molecular mechanisms mediated by different photoreceptors including cryptochromes, phytochromes, and UVR8, integrated with species-specific metabolic networks.

### 4.3 Molecular mechanisms of light quality regulation

Light quality regulation of betalain synthesis begins with precise signal perception by specific photoreceptors: blue light by cryptochromes, red/far-red by phytochromes, and UV-B by UVR8 (Polturak and Aharoni, 2018; Ortega-Hernández et al., 2020). These signals converge on the highly conserved HY5-COP1 module, the central regulatory hub: COP1 targets HY5 for degradation under dark conditions, inhibiting photomorphogenesis; light disrupts COP1/SPA activity, stabilizing HY5 to activate downstream genes. In quinoa seedlings, UV-B activates HY5 accumulation via UVR8, further enhanced by white light pretreatment, indicating HY5 integrates complex light signals.

Stabilized HY5 directly binds cis-acting elements in target gene promoters, achieving precise regulation of key genes in different pigment pathways. In anthocyanin pathways, HY5 activates regulatory (PAP1, MYB10) and structural (DFR, ANS) genes; in carotenoid pathways, it regulates rate-limiting enzymes (PSY, PDS) (Zhao et al., 2022). In betalain synthesis, HY5 upregulates key genes including CYP76AD1, driving L-tyrosine conversion to betalamic acid and promoting betalain biosynthesis (Cá, 2023). Light quality may fine-tune red/yellow pigment ratios through differential regulation of P450 genes (CYP76AD1, CYP76AD5, CYP76AD6) (Sunnadeniya et al., 2016). However, one study suggests that light signals regulate photosynthesis-related genes (RCA, RbcS1A) via HY5/PIFs, with observed betalain changes serving as indirect reporters of HY5/PIFs transcriptional activity rather than direct pathway regulation (Liu et al., 2024).

Within broader regulatory networks, the HY5-COP1 module integrates multiple signals. Crosstalk among photoreceptor pathways (e.g., blue/far-red synergy) may enhance induction. Blue light often promotes reducing sugar accumulation and lowers pH, providing favorable conditions for pigment synthesis. UV light induction is often associated with ROS accumulation, integrating betalain synthesis as an antioxidant into plant stress defense networks (Polturak and Aharoni, 2018). Ultimately, through light-hormone interactions and the HY5-PIFs activator-repressor module, plants achieve precise spatiotemporal control of metabolic gene expression, yielding diverse, species-specific pigment outcomes (Zhao et al., 2010; Ortega-Hernández et al., 2020).

## 5 Genetic and transcriptional regulatory basis of betalain synthesis

### 5.1 Key structural genes in the betalain biosynthetic pathway

Betalain biosynthesis is completed by three core classes of structural genes sharing L-tyrosine as precursor. The first class, CYP76AD family cytochrome P450 enzymes, channel tyrosine into the pathway. This family exhibits functional differentiation: CYP76AD1 (beet R locus) is a bifunctional enzyme with both tyrosine hydroxylase and cyclo-DOPA-forming activity, catalyzing tyrosine→L-DOPA→cyclo-DOPA. It serves as the molecular switch for red betacyanin production; its loss causes red-to-yellow color change (Sunnadeniya et al., 2016). In contrast, CYP76AD5 and CYP76AD6 possess only tyrosine hydroxylase activity, participating only in yellow betaxanthin synthesis and cannot substitute for CYP76AD1 (Barba-Espin et al., 2018).

The second class comprises the DODA family and glycosyltransferases. DODA converts L-DOPA to betalamic acid, the common chromophore precursor, serving as the rate-limiting enzyme. Even with abundant upstream precursors, low DODA efficiency limits production; DODA overexpression significantly enhances yield (Jung and Maeda, 2024). Glycosyltransferases convert unstable intermediates into stable, soluble end-products, critical for efficient accumulation. In *Amaranthus tricolor*, cyclo-DOPA-5-O-glucosyltransferase (*AmcDOPA5GT*) glycosylates cyclo-DOPA to stable cyclo-DOPA-5-O-glucoside, which condenses with betalamic acid to generate red betanin; enzyme absence results in only trace pigment despite normal upstream expression (Chang et al., 2021). Glycosylation significantly enhances chemical stability (betanin about 17× more stable than its aglycone), increases water solubility for vacuolar storage, and creates pigment diversity through different glycosylation patterns and subsequent acylations.

These structural genes exhibit coordinated genomic organization. In dragon fruit, betalain synthesis genes (*CYP76AD-α*, *CYP76AD-β*, *DODA-α*, *5GT*) form an about 12 Mb cluster on chromosome 3, showing coordinated upregulation during fruit ripening (Zheng et al., 2021). Quinoa contains two species-specific *CqCYP76AD-α*, *CqDODA-α* clusters, suggesting spatial proximity enables transcriptional coordination to optimize metabolic flux (Li et al., 2025). Multi-level regulation exists: high-temperature downregulates Amaranthus 5GT, becoming rate-limiting (Li et al., 2025); white bougainvillea bracts result from *BpCYP76AD1* post-transcriptional silencing (Ohno et al., 2021). Evolutionarily, the mono-functional *CYP76AD-β* branch is ancestral, while bifunctional α branch (e.g., *CYP76AD1*) evolved through neofunctionalization, enabling fine regulation of red-yellow pigment ratios (Sunnadeniya et al., 2016).

### 5.2 Identification and functional characterization of core transcription factors

Transcriptional regulation of betalain synthesis is centered on a simplified mode distinct from the anthocyanin-regulating MBW (MYB-bHLH-WD40) complex. The core JA signaling module consists of the F-box protein COI1, JAZ transcriptional repressors, and MYC transcription factors (Chini et al., 2007). MYC2, belonging to the IIIe clade of bHLH transcription factors, serves as the master regulator of JA-responsive gene expression (Song et al., 2022; Luo et al., 2023). Its N-terminus contains a JAZ-interacting domain (JID) and a transcriptional activation domain (TAD) that interacts with the Mediator subunit MED25, while its C-terminal bHLH domain binds G-box (CACGTG) and related cis-elements in target gene promoters (Hou et al., 2023; Zeng et al., 2025).

In the absence of JA, JAZ proteins directly bind MYC2 and recruit the co-repressors NINJA and TOPLESS to silence JA-responsive genes (Chini et al., 2007; Song et al., 2022). Upon stress perception, JA-Ile, the bioactive jasmonate ligand, promotes SCF(COI1)-mediated ubiquitination and 26S proteasomal degradation of JAZ repressors, thereby releasing MYC2 to activate downstream transcription (Hou et al., 2023; Zeng et al., 2025). MYC2 functions redundantly with its close homologs MYC3 and MYC4, forming homo- and heterodimers that coordinately regulate overlapping sets of target genes (Song et al., 2022; Luo et al., 2023).

MYC2 integrates JA signaling with diverse physiological processes, including secondary metabolite biosynthesis, photomorphogenesis, and stress responses (Luo et al., 2023; Zeng et al., 2025). In Arabidopsis, MYC2 and MYC3 directly bind the HY5 promoter to activate its expression, establishing a direct molecular connection between JA and light signaling (Song et al., 2022). Notably, Notably, under certain light conditions, MYC2 and HY5 can exhibit antagonistic regulation, suggesting a complex interplay rather than a simple linear pathway (Song et al., 2022; Luo et al., 2023). In betalain-producing plants, whether MYC2 directly regulates betalain biosynthetic genes or functions through intermediate MYB transcription factors remains to be experimentally determined, representing an important direction for future investigation.

However, in Caryophyllales plants during the transition from anthocyanins to betalains, the MBW regulatory complex underwent repeated degeneration and loss, while MYB factor functions were retained and strengthened. Classical genetic studies designated two beet pigment loci as R and Y; subsequent modern genomics technologies successfully resolved: the R locus corresponds to the key structural gene *CYP76AD1*, while the Y locus encodes an R2R3-MYB transcription factor, *BvMYB1* (Sunnadeniya et al., 2016; Wu et al., 2022). Although *BvMYB1* originated from anthocyanin-regulating MYB branches, its R3 domain contains variant critical bHLH interaction motifs, having lost the ability to interact with bHLH proteins, instead directly activating *CYP76AD1* and *DODA* expression, revealing the simplified regulatory mode of the betalain pathway, termed “MYB-centric, independent of typical MBW complexes” (Lloyd et al., 2017; Wu et al., 2022).

The bHLH transcription factor family also plays important regulatory roles in betalain synthesis, although their mode of action exhibits significant divergence from classical MBW complexes. In dragon fruit, genome-wide identification yielded 165 *HubHLH* genes (Chen et al., 2023a). Among these, *HubHLH159* is highly expressed during red-fleshed dragon fruit ripening, localizes to the nucleus, possesses transcriptional activation activity, and directly activates transcription of structural genes (*HuADH1*, *HuCYP76AD1-1*, *HuDODA1*) by specifically binding G-box elements in their promoters (Chen et al., 2023a). Further research revealed that *HpbHLH48* and *HpbHLH64* are also indispensable positive regulators (Cai et al., 2025). Their mechanisms exhibit differentiation: HpbHLH48 possesses transcriptional activation activity and directly binds E-box elements in the HpADH1, HpCYP76AD1-1, and HpDODA1 promoters to activate their expression. In contrast,

HpbHLH64 binds the HpDODA1 and HpB5GT5 promoters but lacks transcriptional activation activity, suggesting that it may require interaction with other co-factors to function. Yeast two-hybrid assays indicate both can form homodimers but exhibit no interactions with each other or with HpbHLH159, revealing functional differentiation mechanisms of bHLH transcription factors in dragon fruit betalain synthesis.

Light and hormone signals deeply participate in the regulatory network of betalain synthesis through key transcription factors HY5 and MYC2. HY5, as a bZIP family transcription factor, serves as a core integration node in light signaling pathways (Cluis et al., 2004). In betalain synthesis pathways, light signals (particularly UV-B) inhibit COP1 E3 ubiquitin ligase activity through UVR8, thereby stabilizing HY5 protein and promoting its nuclear accumulation. HY5 activates expression of betalain structural genes (such as *CYP76AD1*, *DODA*) by directly binding G-box (CCACGTG) elements in their promoters (Tossi et al., 2021). This regulatory mechanism is highly conserved with anthocyanin pathways, although HY5 may coordinate with MYB transcription factors (such as *BvMYB1*) to form regulatory modules while subject to cross-regulation by hormone signals including cytokinins and jasmonates (Tossi et al., 2021). MYC2, as the core bHLH transcription factor in jasmonate signaling pathways, regulates MBW complex activity in anthocyanin models through integration of jasmonate signals. Recent studies revealed that MYC2 directly binds G-box/PBE elements in the HY5 promoter to activate its transcription, directly linking jasmonate and light signaling pathways at the HY5 level (Chakraborty et al., 2019; Ortigosa et al., 2020). In Arabidopsis, MYC2 mediates crosstalk between JA and other hormone pathways, including GA, ET, and ABA, through interactions with DELLA proteins, EIN3/EIL1, and other signaling components (Song et al., 2022; Luo et al., 2023). Whether these multi-hormone interactions converge with light signaling to regulate betalain biosynthesis in Caryophyllales remains an open question. Given the evolutionary relationship between anthocyanin and betalain pathways and the conserved nature of JA and light signaling, it is plausible that MYC2 may function through similar mechanisms in betalain-producing species; however, direct experimental evidence is currently lacking.

### 5.3 Coordinated regulatory networks of transcription factors

The transcriptional regulatory network of betalain synthesis exhibits organizational patterns distinctly different from anthocyanin pathways. In anthocyanin models, the highly conserved MBW ternary complex (MYB-bHLH-WD40) serves as the core regulatory unit, with bHLH factors acting as “bridges” connecting MYB and WD40 proteins, coordinately activating downstream structural gene expression (Chen et al., 2023b). However, in betalain pathways, this classical complex regulatory mode has undergone significant simplification. Evolutionary analyses indicate that in Caryophyllales plants transitioning from anthocyanins to betalains, MBW regulatory complexes have undergone repeated, lineage-specific degeneration in betalain-producing Caryophyllales. Genomic analyses across 357 species revealed that key bHLH partners of the anthocyanin MBW complex, specifically GL3/EGL3 and MYC1 orthologs, are frequently absent from betalain-pigmented lineages. For example, the GL3 ortholog has been completely lost in *Beta vulgaris* (Pucker et al., 2024). In contrast, the WD40 protein TTG1 is universally retained, and certain R2R3-MYB factors have been functionally repurposed to directly activate betalain structural genes without requiring bHLH partners. This bHLH-independent activation was experimentally demonstrated by yeast one-hybrid assays showing that BvMYB1 (PAP1a) binds and activates CYP76AD1 and DODA promoters without bHLH interaction (Hatlestad et al., 2015; Pucker et al., 2024). Studies in *Amaranthus tricolor* provide direct evidence: *AtrMYB72* directly binds MBS elements in the *AtrCYP76AD1* promoter to activate its transcription (Xue et al., 2025). In brief, MYB regulation of anthocyanins represents spatiotemporal “teamwork,” while its regulation of betalains represents functional switching to “individual action”.

Although MBW complexes are incomplete in betalain pathways, other transcription factors including bHLH participate in betalain synthesis coordinated regulation through independent or alternative pathways, forming multi-level integration with environmental signals. In dragon fruit, *HubHLH159* significantly activates expression of betalain synthesis key genes *HuADH1* and *HuDODA1* through direct binding to G-box elements in their promoters (Chen et al., 2023a). Additionally, the R3 domain of *HuMYB1* contains a highly conserved bHLH interaction motif, suggesting potential for interaction with bHLH factors (Xie et al., 2021). Environmental signals further enrich this network’s complexity: light indirectly drives betalain production through transcription factors HY5 (activator) and PIFs (repressor) acting on G-box and I-box elements in promoters (Liu et al., 2024). The core factor of jasmonate signaling pathways, MYC2, may also participate in regulation through signal crosstalk (Ghorbel et al., 2021; Tian et al., 2024). This flexible coordinated network, centered on MYB, with bHLH alternative participation, and integrating multiple environmental signals, achieves fine spatiotemporal regulation of betalain accumulation.

## 6 Signal crosstalk between MeJA and light quality

### 6.1 Synergistic regulation by MeJA and light quality

Plants integrate endogenous hormone signals with environmental cues to coordinate growth, development and defence metabolism (Ghorbel et al., 2021; Ma et al., 2025). Studies have shown that MeJA not only enhances plant resistance to pathogens by activating pathogenesis-related (PR) protein expression (Ji et al., 2021b) but also alleviates damage from abiotic stresses such as salt and drought by regulating ion balance, enhancing antioxidant enzyme activities, and increasing osmolyte content (Rao et al., 2022; Shang et al., 2024).

Light quality, perceived by photoreceptors (phytochromes, cryptochromes, UVR8), regulates both photomorphogenesis and secondary metabolism (Ouzounis et al., 2015). Different light qualities act as precise “metabolic switches”: red light activates phenylpropanoid genes, promoting flavonoids and anthocyanins (Wang et al., 2022); blue light stabilizes HY5 to regulate anthocyanin genes (Li et al., 2021b); UV-B upregulates phenylpropanoid enzymes via UVR8-HY5 (Mannucci et al., 2022). These photoreceptor systems form regulatory hubs through shared signaling components, achieving precise spatiotemporal control of plant metabolism.

Critically, MeJA and photoreceptor-mediated signaling pathways are not independent; they exhibit extensive crosstalk and synergistic interactions. Red light, perceived by phytochrome B (phyB), promotes the degradation of PIF4. Under low R:FR light conditions, stabilized PIF4 activates the transcription of a sulfotransferase (STa) that inactivates JA precursors, thereby reducing JA-Ile availability. Red light also enhances JA-Ile accumulation by promoting COI1-dependent degradation of JAZ repressors (Hong et al., 2024). Blue light stabilizes HY5 via cryptochrome 1, synergistically upregulating phenylpropanoid gene expression with MYC2 from the JA pathway (Verma et al., 2021). This integration of hormone and light signals enables plants to mount more sensitive and efficient metabolic responses to environmental changes, maximizing survival benefits. Elucidating how MeJA and light quality coordinately regulate secondary metabolism, particularly using betalain synthesis as a paradigm, has become a research frontier. This review systematically examines the independent and synergistic regulation of secondary metabolism by light and MeJA, focusing on signal integration and transcriptional crosstalk, to provide new perspectives for understanding plant environmental adaptation and crop quality improvement.

### 6.2 Physiological evidence for MeJA-light quality interactions

Physiological evidence for MeJA-light quality interactions first manifests in their synergistic inductive effects on secondary metabolites. In various plant systems, combined light and jasmonate signal treatments produce additive or synergistic effects exceeding single treatments: red light combined with MeJA significantly enhances stilbene compound induction; blue light with MeJA synergistically promotes polyphenol accumulation through upregulation of key polyphenol biosynthetic gene expression (Dong et al., 2025); under balanced red-green-blue light backgrounds, MeJA treatment concentration-dependently significantly increases mint anthocyanin content, although this promotion exhibits pathway specificity, with high MeJA concentrations inhibiting total phenol content while promoting anthocyanin accumulation (Dangsamer et al., 2025). This evidence indicates that light quality signals and MeJA signals converge and integrate at the transcription factor level (such as MYB, bHLH), enabling plants to finely regulate secondary metabolite biosynthesis direction based on light environment changes (Kazan and Manners, 2011).

Apple fruit coloration studies provide more direct physiological evidence. In ‘Fuji’ apples, MeJA alone hardly induced peel coloration, but combined UV-B+MeJA for 72 h increased anthocyanin content about 20-fold in immature fruit and ~4-fold in color-turning fruit (Ryu et al., 2022). At the molecular level, combined treatment significantly upregulated anthocyanin pathway genes: transcription factor MdMYB10 (95-fold) and structural genes MdCHS (45-fold), MdLDOX (14-fold), and MdUFGT (22-fold), while MeJA alone hardly induced these genes, confirming that synergistic activation at the transcriptional level underlies efficient anthocyanin accumulation (Ryu et al., 2022).

Light-MeJA interactions also reshape plant phenotypes through coordinated regulation of photosynthesis, carbon-nitrogen metabolism, and development. Appropriate MeJA concentrations with specific light qualities optimize stomatal aperture and photosynthetic enzyme activities, while high MeJA induces stomatal closure and inhibits photosynthesis. Their interaction affects source-sink relationships: decreased leaf sugar accumulation but increased fruit sugar and organic acid content, indicating enhanced photosynthate transport to reproductive organs. Low MeJA with light promotes reproductive growth, while high MeJA inhibits flower bud differentiation, reflecting growth-defense trade-offs. Additionally, their synergy enhances pest resistance and differentially regulates mineral nutrient uptake, achieving dynamic balance between growth promotion and defense activation (Adrian et al., 2025).

In betalain research, direct evidence for MeJA-light quality interactions is limited. However, combinations of MeJA with other stress factors significantly enhance betalain production, suggesting similar crosstalk mechanisms may exist. Future research should systematically evaluate different light quality and MeJA combinations on betalain accumulation and elucidate their signal integration mechanisms at the molecular level.

### 6.3 Molecular mechanisms of crosstalk

The molecular crosstalk between MeJA signals and light quality signals centers on convergence of key transcription factors from both pathways on target gene promoters and direct protein-protein interactions. In light signal transduction, following photoreceptor (e.g., phyB, cry) perception of light quality changes, downstream responses are regulated on one hand through HY1 (affecting phytochrome chromophore synthesis); on the other hand, COP1, a key suppressor in light signaling, may indirectly affect JAZ protein stability through unknown mechanisms, but direct degradation has not been demonstrated (Dangsamer et al., 2025). Genetic analysis indicates that in light signaling pathways, HY1 exhibits epistasis over MYC2, meaning HY1 acts downstream of MYC2 to regulate light responses; while in jasmonate signaling pathways, MYC2 positively regulates JA responses downstream of HY1. This dynamic hierarchical relationship reflects functional switching of identical components in different signaling pathways (Prasad et al., 2012).

MeJA signal enhancement accelerates and amplifies light signal transduction, with the molecular basis lying in integrated activation of light and jasmonate signals at the transcription factor MYB level. Light signals initiate downstream signaling pathways through photoreceptors, providing basal transcriptional activity for anthocyanin synthesis; MeJA, through relieving JAZ protein-mediated repression of MYC2, activates jasmonate-responsive elements. Both coordinately regulate MYB promoter regions, elevating MYB expression and thereby substantially upregulating anthocyanin synthesis structural genes, ultimately driving efficient anthocyanin accumulation (Ryu et al., 2022).

At the molecular level, MYC2 (MeJA signaling) and HY5 (light signaling) serve as core nodes integrating multiple environmental signals. ChIP-seq and EMSA confirm that MYC2 and MYC3 directly bind PBE elements in the HY5 promoter to activate its transcription; HY5 transcript and protein levels significantly decrease in myc mutants, providing direct evidence that MYC promotes photomorphogenesis by activating HY5 expression (Chakraborty et al., 2019; Ortigosa et al., 2020).

The molecular mechanism of MeJA-light co-action is also reflected in synergistic activation of secondary metabolic pathways: integration of light and jasmonate signals at the transcription factor level leads to coordinated activation of phenylpropanoid (PAL), terpenoid (TPS), and fatty acid metabolism, promoting accumulation of defensive secondary metabolites such as phenolics and terpenoids (Adrian et al., 2025).

However, in betalain systems, MeJA regulation exhibits interspecific differences: in *A. philoxeroides*, MeJA upregulates betalain synthesis genes and increases tyrosine content; while in *A. sessilis*, despite increased betalain content, gene expression is suppressed, suggesting high tyrosine-mediated negative feedback inhibition and uncoupling between gene expression and metabolite accumulation (Milech et al., 2023).

### 6.4 Construction of multi-signal coordinated regulatory network

Integrating the evidence summarized above, we propose an integrated model of MeJA and light quality signal crosstalk regulating betalain biosynthesis (**Figure 1**). **Figure 1** presents an integrated model of MeJA and light quality signal crosstalk converging on betalain biosynthetic genes. In this model, light quality signals are perceived by specific photoreceptors (UVR8, CRY1/CRY2, and PHYB) and converge on the COP1/SPA complex. Light disrupts COP1/SPA activity, thereby relieving COP1-mediated ubiquitination and degradation of HY5, allowing its nuclear accumulation. On the jasmonate side, exogenous MeJA is first demethylated to JA and subsequently conjugated to isoleucine by JAR1 to form the bioactive ligand JA-Ile. JA-Ile promotes SCF(COI1)-mediated ubiquitination and proteasomal degradation of JAZ transcriptional repressors, thereby releasing MYC2 and MYC3 from repression.

At the transcriptional integration level, MYC2 and MYC3 directly bind PBE elements (CATGTG) in the HY5 promoter, as demonstrated by ChIP-seq and EMSA in *Arabidopsis* (Ortigosa et al., 2020). A potential feedback loop in which HY5 regulates MYC2/MYC3 expression is hypothesized but awaits experimental validation. Both HY5 and MYC2/MYC3 signals converge on R2R3-MYB transcription factors, which bind MBS (MYB-binding site) elements in the promoters of the key structural genes CYP76AD1 and DODA. Direct binding of MYB factors to these promoters has been confirmed by yeast one-hybrid assays; for DODA, direct regulation was further supported by cycloheximide-resistant transcriptional activation (Hatlestad et al., 2015; Xue et al., 2025).

Downstream of transcriptional activation, the betalain biosynthetic pathway proceeds through four key enzymatic steps: ADH provides the tyrosine precursor; CYP76AD1, encoding a bifunctional cytochrome P450, catalyzes the conversion of L-tyrosine to L-DOPA and subsequently to cyclo-DOPA (Sunnadeniya et al., 2016); DODA catalyzes the formation of betalamic acid from L-DOPA; and cDOPA5GT glycosylates cyclo-DOPA for stable betacyanin production (Xie et al., 2021). The coordinated action of these enzymes ultimately drives betalain pigment accumulation.

Solid arrows in **Figure 1** indicate experimentally supported regulatory relationships; dashed arrows represent hypothesized interactions; the inhibition symbol (⊥) denotes protein degradation or transcriptional repression. While the core architecture of this model is supported by experimental evidence, several aspects remain to be validated. MYC2/MYC3-HY5 promoter binding has been confirmed only in *Arabidopsis*, and direct evidence for this interaction in betalain-producing Caryophyllales species is currently lacking. Furthermore, whether the MYC2-HY5-MYB regulatory logic is conserved across different betalain-producing species, and how signal integration varies with different light qualities, developmental stages, and tissue types, requires further investigation.

## 7 Conclusion

Betalain biosynthesis operates through a simplified regulatory architecture in which R2R3-MYB transcription factors activate structural genes independently of the canonical MBW (MYB-bHLH-WD40) complex. In Arabidopsis, MYC2 and MYC3 directly bind PBE elements in the HY5 promoter and activate its transcription, establishing a molecular link between jasmonate and light signalling pathways (Ortigosa et al., 2020). Both pathways are proposed to converge on R2R3-MYB transcription factors, which recognize MBS motifs in the CYP76AD1 and DODA promoters (Hatlestad et al., 2015; Xue et al., 2025). Downstream of this transcriptional convergence, four principal enzymes catalyse the conversion of tyrosine to betacyanins and betaxanthins: ADH, CYP76AD1, DODA and cDOPA5GT (Sunnadeniya et al., 2016; Xie et al., 2021).

Integrating the evidence summarized in this review, we propose a MYC2-HY5-MYB signal integration model (**Figure 1**) in which jasmonate and light signals coordinately regulate MYB-dependent betalain gene expression. In this model, solid lines represent experimentally supported regulatory relationships, dashed lines indicate hypothesized interactions, and T-bars denote protein degradation or transcriptional repression. Several components of this model await experimental validation in betalain-producing species. Direct evidence for MYC2-HY5 physical or functional interactions in Caryophyllales is currently lacking. The evolutionary conservation of this regulatory logic across betalain-producing lineages has not been systematically examined. Furthermore, how signal integration varies with specific light qualities, developmental stages and tissue types remains unexplored. Addressing these questions through multi-omics approaches combined with targeted gene functional studies in *Beta vulgaris*, *Amaranthus tricolor* and *Hylocereus* spp. will be essential to resolve the regulatory architecture of betalain biosynthesis and to translate this understanding into biotechnological strategies for pigment production.
